# The Gut Microbiota in Cardiovascular Disease and Arterial Thrombosis

**DOI:** 10.3390/microorganisms7120691

**Published:** 2019-12-13

**Authors:** Anna Lässiger-Herfurth, Giulia Pontarollo, Alexandra Grill, Christoph Reinhardt

**Affiliations:** 1 Center for Thrombosis and Hemostasis (CTH), University Medical Center Mainz, Johannes Gutenberg University of Mainz, Langenbeckstrasse 1, 55131 Mainz, Germany; a.herfurth@uni-mainz.de (A.L.-H.); giulia.pontarollo@unimedizin-mainz.de (G.P.); alexandra.grill@uni-mainz.de (A.G.); 2 German Center for Cardiovascular Research (DZHK), Partner Site RheinMain, 55131 Mainz, Germany

**Keywords:** gut microbiota, vascular inflammation, arterial thrombosis, cardiovascular disease, blood pressure regulation

## Abstract

The gut microbiota has emerged as a contributing factor in the development of atherosclerosis and arterial thrombosis. Metabolites from the gut microbiota, such as trimethylamine N-oxide and short chain fatty acids, were identified as messengers that induce cell type-specific signaling mechanisms and immune reactions in the host vasculature, impacting the development of cardiovascular diseases. In addition, microbial-associated molecular patterns drive atherogenesis and the microbiota was recently demonstrated to promote arterial thrombosis through Toll-like receptor signaling. Furthermore, by the use of germ-free mouse models, the presence of a gut microbiota was shown to influence the synthesis of endothelial adhesion molecules. Hence, the gut microbiota is increasingly being recognized as an influencing factor of arterial thrombosis and attempts of dietary pre- or probiotic modulation of the commensal microbiota, to reduce cardiovascular risk, are becoming increasingly significant.

## 1. Introduction

The human body surface is host to one of the most diverse microbial ecosystems in nature—the commensal microbiota [1]. In particular, the impact of the gut microbiota on host physiology is moving more and more into the focus of biomedical research [2]. This densely colonized microbial ecosystem critically influences the host’s immune homeostasis via microbial-associated molecular patterns (MAMPs) and through the signaling of active metabolites [3,4]. For example, stimulation with MAMPs increases the phagocytic capacity and the response to cytokines of macrophages and neutrophils and drives atherogenesis [5,6,7,8]. In contrast, metabolites such as short chain fatty acids (SCFAs) inhibit interferon-γ (IFN-γ) production and protect from mucosal inflammation, but also reduce the development of atherosclerotic lesions [5,6].

The gut microbiota influences the development of vascular inflammation and atherosclerosis [7,8,9]. Various mouse models have shown that intestinal microbial communities have a crucial influence on vascular inflammatory phenotypes, the development of cardiovascular diseases (CVD), and arterial thrombosis [10]. While interfering with host energy metabolism [8], the gut microbiota impacts on the development of atherosclerosis, as demonstrated by the association-based sequencing studies on patient samples [8,11,12,13] and by the depletion of the gut microbiota with antibiotics or by comparing germ-free (GF) with conventionally raised (CONV-R) mouse atherosclerosis models [7,13,14,15,16]. Via the activation of pattern recognition receptors (PRRs) on platelets and endothelial cells, MAMPs promote arterial thrombosis and stimulate the synthesis of prothrombotic von Willebrand factor (VWF) in the hepatic endothelium, along with increased FVIII plasma levels [9,17]. Thus, the commensal gut microbiota is increasingly recognized as an environmental factor that contributes to arterial thrombosis.

In addition to vascular inflammatory phenotypes, MAMPs such as lipopolysaccharides (LPS) and lipoteichoic acid (LTA) [18], which increase in the bloodstream after fat-rich meals, drive metabolic inflammation [19,20,21]. The gut inflammatory response is modulated through microbiota-derived MAMPs, that signal via Toll-like-receptors (TLRs) and nucleotide-binding oligomerization domain-like receptors (NOD-like receptors) [22,23,24,25]. Interestingly, the expression of TLRs is influenced by their adaptor proteins [22,23,24,25]. Work with GF mouse models revealed that the gut microbiota promotes diet-induced obesity [7,26,27]. This is achieved by a number of mechanisms, such as modulating the appetite via decreased leptin sensitivity, influencing gut motility and uptake, storage, and expenditure of energy [28]. Metabolites of microbial origin are not only direct energy sources, but also function as messengers, such as trimethylamine (TMA) or SCFAs [29]. An altered gut microbiota results in an imbalance of energy consumption and weight gain, as shown in obese individuals, followed by an increased risk of cardiometabolic disease onset [29,30,31,32].

With the fast advancement in gnotobiotic research, microbe-host interactions are analyzed by comparing GF mouse models with their conventionally raised (CONV-R) counterparts [2]. Through monocolonization studies, the impact of single strains of bacteria can be analyzed. Antibiotic depletion of the microbiota, as a standard method for the investigation of bacterial influence, is a complementary approach, but less unequivocal due to the various side effects of antibiotics [33]. In addition to the well-standardized gnotobiotic animal models, 16S rRNA sequencing is widely used to identify the diversity of bacteria and the bacterial taxonomy of microbiomes. This is an effective way to catalogue specific taxa, as presently only 20% of the microbiota can be cultivated and the human gut hosts approximately 3.9 × 10^13^ bacteria [34,35]. Studies showed large differences in the abundance and variety of bacterial taxa in the gastrointestinal tract, which are associated with diet-induced obesity in comparison to healthy control groups [20,36]. This opens up the diagnostic possibility of defining bacterial taxa that might be useful to predict individual cardiometabolic disease risk [13]. Due to the rapid advances in 16S rRNA sequencing, chances are provided to pharmacologically target the gut microbiota more precisely, through selective inhibition with antibiotics, stimulating or suppressing the development of specific taxa with pro or prebiotics, or even by non-lethal, selective inhibition of microbial metabolic enzyme functions [13]. Identifying microbial enzymes or transplanting fecal samples from healthy to diseased individuals could be a future strategy to prevent the development of CVD [37,38]. However, in spite of the recent advances that linked the gut microbiota with the development of cardiometabolic disease and arterial thrombosis, the gaps in knowledge about the interaction between the human immune system, the gut microbiota, and the development of these age-dependent disease states are still big.

Here, we provide an overview of the functional impact of the gut microbiota, influencing vascular inflammation, on the development of atherosclerotic lesions and arterial thrombosis. This review puts its focus on gut microbiota-derived MAMPs and metabolites, which play a key role in shaping the inflammatory response, orchestrating immune homeostasis, and contributing to CVD risk and arterial thrombosis. In this review, we summarize recent studies providing insights into the emerging link between the gut microibota and arterial thrombosis.

## 2. Impact of the Gut Microbiota on Vascular Inflammation and Blood Pressure Regulation

Hypertension, as well as type 2 diabetes, obesity, and cardiovascular diseases, are common health problems in ageing populations of Western societies [39]. Evidence about gut microbiota influencing blood pressure is accumulating [40]. Many different models inducing hypertension are used in studies, and in all of them, a correlation with an altered gut microbiota could be noted [41]. Blood pressure in healthy subjects is regulated via the renin-angiotensin-aldosteron-system (RAAS), the immune system, endothelial function, and the sympathetic nervous system [41,42,43]. By inducing hypertension in a chronic angiotensin II-infusion model via implanted osmotic minipumps in mice, 12 metabolites in plasma and 86 metabolites in feces were detected to change under angiotensin II treatment [41]. The study compared wild-type, conventionally raised mice with GF mice, and demonstrated that the altered metabolites detected in CONV-R mice showed no significant changes in the GF group [41]. This evidence proves that gut microbiota affects the RAAS function and related metabolites [44]. In addition, the hypertensive phenotype was transferred via fecal transplantation into GF mice repeatedly, proving an influence of the gut microbiota on blood pressure [44]. Various approaches suggest that microbiota metabolites interact with receptors in the brain and vascular walls, as well as affecting immune cells and changing blood pressure [45,46].

It was demonstrated that the composition of the gut microbiota impacts the balance of T-cells (regulatory T cells vs Th17 cells), thus affecting the development of hypertension [47]. Tissue infiltration by T-cells causes higher levels of chemokines and cytokines, which recruit immune cells to the site of inflammation [42,47,48]. Predominant cytokines in hypertensic patients are IFN-γ, TNF-α, and IL-17A, all being influenced by the presence of MAMPs derived from the gut microbiota [47].

Altered gut microbiota was identified in patients with hypertension, accompanied with increased blood LPS levels and a gut microbiome with decreased capacity for butyrate production [49]. The protective role of butyrate and acetate producing microbiota was demonstrated in a microbiome study with Wistar Kyoto rats and spontaneously hypertensive rats [50]. This underlines a protective role of butyrate in case of hypertension and blood pressure regulation. Interestingly, additional microbiota-regulated metabolic pathways exist that can affect vascular function, e.g., the serotonin biosynthesis pathway and the conversion of choline to TMA [51,52]. Butyrate seems to protect patients from increased blood pressure, cardiac hypertrophy, renal injury, and fibrosis [53].

Recent studies identified receptors that interact with specific metabolites derived from the microbiota, which play important roles in blood pressure regulation. “Metabolite-sensing” G-coupled protein receptors (GPCRs) and olfactory receptor 78 (Olfr78) are expressed in different organs and especially GPCRs 41 and 43 in renal and vascular tissue are activated through SCFAs, regulating blood pressure [47]. Propionate, for example, triggers blood pressure changes through Olfr78 in smooth muscle cells of arteries and in autonomic nerves in the heart, kidney, and gut [54]. On one hand, it increases renin levels causing higher blood pressure, but on the other hand, decreases blood pressure via GPR 41 [54]. This evidence demonstrates how subtle the systemic regulation of blood pressure is and how dependent it is on the site and kind of receptor expression. In conclusion, gut microbiota influences and even regulates blood pressure through various pathways, especially through microbial derived metabolites.

## 3. Patterns and Metabolites from the Gut Microbiota as Drivers of Atherosclerosis and Arterial Thrombosis

The intestinal microbiota is separated from the host by the mucus layer and a specializedepithelial lining [55,56]. The epithelium has to fulfill very specific requirements to protect the host from foreign antigens, but at the same time, allow substances to enter the portal circulation for energy metabolism and adequate nutrient supply [57]. Not only the epithelial barrier, but also the gut-vascular barrier (GVB), needs to reach up to these requirements [58,59,60]. For instance, in the lacteal microvasculature, the integrity of the GVB is an important determinant of dietary lipid uptake [61]. Interestingly, the gut microbiota not only affects the permeability of the intestinal vasculature, but it is also able to trigger the formation of intricate capillary networks and lacteals in small intestinal villus structures, which serve as nutrient uptake [62,63,64,65]. Diet influences intestinal permeability and the uptake of microbiota-derived molecules [20]. Metabolites provoke immune activation and low-grade inflammation, thus modifying the transcription of genes that influence host energy metabolism [21,27,66].

One of the most impactful microbiota-derived patterns that interfere with the human organ functions is lipopolysaccharide (LPS) [67]. LPS is an integral constituent of the outer membrane of Gram-negative bacteria [55]. LPS, as one of the various MAMPs, gives signals through the pattern recognition receptor (PRR) Toll-like-receptor-4 (TLR4) [68]. In humans, more than 13 TLRs, which are functionally expressed on many different cell types, initiate host responses to MAMPs [69]. In the gut epithelium, LPS from bacteria signals through TLR4 [70,71]. Importantly, excessive TLR4 signaling is prevented by endotoxin tolerance, through the down-regulation of TLR4 signaling components in the intestinal epithelium after birth [72].

There is increasing evidence for the gut microbiota as a relevant source of MAMPs, contributing to low but metabolically active levels of these molecules in the bloodstream [21,73]. Dependent on gut barrier function, these blood-borne MAMPs may contribute to remote signaling in distant organs [21,74] and promote chronic inflammatory processes, such as white adipose tissue inflammation, atherosclerosis, and cerebral cavernous malformations [11,74,75,76]. The presence of a gut microbiota, constantly challenging the host, drives the expression of inflammatory mediators, which then recruit immune cells. Among all, LPS has been demonstrated to contribute to metabolic inflammatory phenotypes [21,77]. In addition, microbiota-derived compounds drive steady-state granulopoiesis and neutrophil ageing, influencing the bone marrow myeloid pool size [3,4]. It was shown that fatty diets induce an increase in blood LPS levels and higher formation of adipocyte precursor cells, which then result in higher risks of vascular diseases and chronic inflammation [78,79]. Thus, microbiota-derived LPS can trigger a vicious circle of inflammatory responses and promote metabolic endotoxemia [80].

Next to LPS, trimethylamine N-oxide (TMAO), a choline metabolite, which was associated with inflammation, atherosclerotic lesion progression, and arterial thrombosis, belongs to the group of signaling active metabolites, that are derived from the gut microbiota [7,12]. Red meat, egg yolk, and fat-rich products contain a high amount of L-carnitine and phosphatidylcholine [13,81]. These two molecules are processed by bacterial trimethylamine (TMA)-lyases from the gut microbiota to trimethylamine (TMA) [82,83,84]. On note, this reaction can only happen when the transport capacity of choline and L-carnitine in the small intestine exceeds [84]. Therefore, flavin-containing monooxygenases in the liver oxidize TMA to TMAO [84], which is linked to increased insulin resistance, as higher TMAO blood levels were noted in diabetes [85]. In addition, TMAO affects chronically infused angiotensin II signaling, contributing to prolonged hypertension [86] and increases the risk of CVD and vascular wall inflammation [44,87].

Interestingly, plasma L-carnitine levels were identified as a predictor of cardiovascular risk in coronary artery disease and peripheral artery disease patients and both dietary choline and TMAO supplementation enhanced atherosclerotic lesion development in atherosclerosis-prone *Apoe*^−/−^ C57BL/6J mice [12,13]. Of note, this was not found in studies with GF *Apoe*^−/−^ mice [14]. While choline supplemented diet augmented atherosclerosis and plaque macrophage content in this mouse atherosclerosis study, broad-spectrum antibiotic treatment (0.5 g/L vancomycin, 1 g/L neomycin sulfate, 1 g/L metronidazole, 1 g/L ampicillin) via the drinking water demonstrated that depletion of the microbiota decreased the choline-dependent enhancement of atherosclerotic lesions in male and female *Apoe*^−/−^ C57BL/6J mice, when kept on a choline-enriched diet at the age of four-weeks until the age of twenty-weeks [16]. Non-lethal inhibition of gut bacterial TMA lyases with 3,3-dimethyl-1-butanol prevented atherogenesis in *Apoe*^−/−^ mice on choline rich diet [10,13]. Furthermore, the microbiome-dependent impact of TMAO on prothrombotic platelet function and arterial thrombosis was demonstrated to be transmissible by fecal transplantation from human donors with low or high TMAO-producing microbiota into GF recipient mice [10,88]. Future clinical studies and experimental research need to address the mechanisms through which TMAO influences CVD and arterial thrombosis.

**Figure 1:**
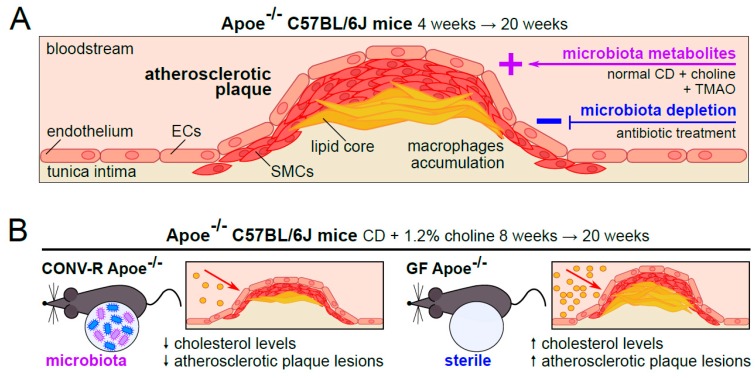
Atherosclerosis in antibiotic-treated mice and in the germ-free *Apoe*-deficient mouse model. (**A**) Pro-atherogenic effects are described for the choline-rich diet and the microbiota-derived choline-metabolite trimethylamine N-oxide (TMAO) [12,13,16]. (**B**) GF *Apoe*-deficient mice on a chow-diet developed an increased lesion size compared to CONV-R *Apoe*-deficient mice and the gut microbiota had a cholesterol-lowering function under chow-diet conditions [9,10,14,15].

While the presence of a gut microbiota resulted in reduced serum cholesterol levels under low-cholesterol feeding conditions, this effect was abolished on a cholesterol-rich diet in the low-density lipoprotein receptor-deficient mouse model (*Ldlr*^−/−^). In CONV-R *Ldlr*^−/−^ mice, no microbiota-dependent effects were observed on late atherosclerotic lesion size on a γ-irradiated cholesterol-rich diet at sixteen-weeks of Western diet feeding with respect to GF counterparts [48]. In accordance to this recent work, Lindskog Jonsson et al. did not find a correlation between plasma TMAO concentration and atherosclerotic lesion size in the aortic root and the relative aortic root lesion size was not significantly different in GF *Apoe*^−/−^ C57BL/6J mice, compared to CONV-R *Apoe*^−/−^ C57BL/6J mice treated with a Western diet [9,14]. A previous study on GF *Apoe*^−/−^ C57BL/6 mice kept on a chow diet reported reduced relative and absolute aortic root plaque areas and reduced macrophage plaque content at twenty-weeks of age [15] (Figure 1). Considering the different outcomes of different studies on atherogenesis, analyzing GF and antibiotic-treated atherosclerosis mouse models from different mouse husbandries, it can be concluded that the microbiota-dependent impact on atherogenesis is strongly influenced by different diets, feeding regime, housing conditions, and the analyzed time point (Table 1). Therefore, additional gnotobiotic research under well-standardized conditions, which relate to published work, is required to resolve the impact of the gut microbiota as a chronic influencing factor in atherosclerotic lesion development, which causes atherothrombotic diseases.

Short chain fatty acids represent another example of microbiota-derived metabolites, having a beneficial influence on cardiometabolic health. Specific bacteria in the large intestine produce SCFAs (i.e., acetate, propionate and butyrate) [89]. Their substrate are non-digestible carbohydrates from plant and fiber-rich diets [89,90]. SCFAs are found in their highest concentrations in the caecum with approximately 130 mmol/kg, and this concentration correlates with that of the portal vein [91]. Gut passage, diet, and gut microbiota composition influence the synthesis of SCFAs [92]. After absorption by the gut epithelium, butyrate is mainly used as an energy source by the epithelium itself, propionate is transported to the liver and converted to sugar and fat [93], and acetate is primarily metabolized in the heart, nervous system, and skeletal muscle. This indicates a different utilization of the three SCFAs in different metabolic processes of various tissues.

## 4. Evidence Linking the Microbiota with CVD and Arterial Thrombosis

Numerous studies have linked the composition of the gut microbiota and its metabolic capacity with CVD risk. For instance, Kelly and coworkers analyzed the microbiota from patients suffering from hearth disorders and linked it with their CVD risk [94]. In this study, seven microbial genera were linked to an increased CVD risk, i.e., Alloprevotella, Prevotella, and Paraprevotella (all three belonging to Bacteroidetes phylum), and Tyzzerella 4, Tyzzerrella, Megamonas, and Catenibacterium (belonging to Firmicutes phylum) [94]. Furthermore, elevated blood levels of TMAO were associated with an increased risk of CVD. In a shotgun sequencing study on fecal samples from patients with symptomatic atherosclerotic plaques (carotid endarterectomy for minor ischemic stroke, transient ischemic attack or amaurosis fugax), the genus *Collinsella* was enriched, whereas *Roseburia* and *Eubacterium* were most abundant in healthy controls [11]. In this study, the characterization of the metagenome revealed an enrichment of genes encoding for the peptidoglycan synthesis pathway. Interestingly, TMAO-producing bacteria were associated with arterial thrombotic risk and could be identified through 16S RNA sequencing analyses [11]. These studies imply that, in the future, the detection of certain indicator species, combined with metagenomics analyses, could become a valuable diagnostic tool in cardiovascular risk assessment and prevention of arterial thrombosis.

**Figure 2:**
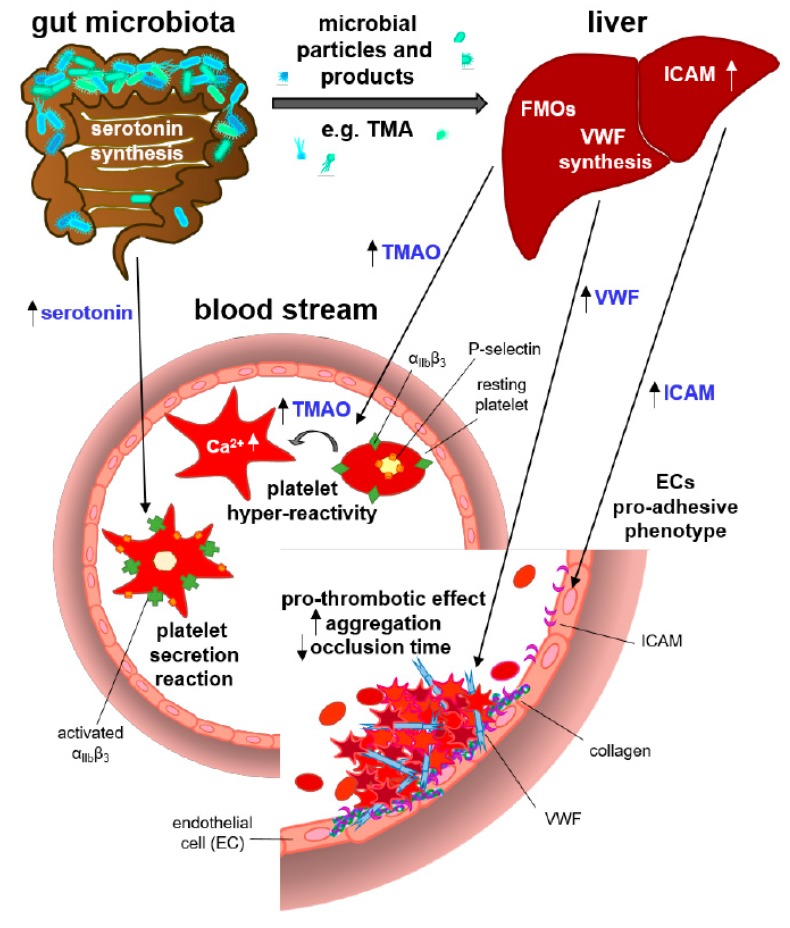
Evidence for a pro-thrombotic role of the gut microbiota as identified by gnotobiotic mouse models. Serotonin from enterochromaffin cells in the intestinal epithelial lining and the microbiota-derived choline-metabolite trimethylamine N-oxide (TMAO) were described to promote platelet reactivity, following agonist-induced platelet activation [82,88]. The gut microbiota increases the expression of endothelial adhesion molecules, such as ICAM-1 and VWF [95,96].

Taking advantage of GF mouse isolator technology, distinct microbiota-host interactions that promote arterial thrombosis in experimental mouse thrombosis models have been identified [17] (Figure 2). Yano et al. unraveled that the commensal microbiota augments the serotonin biosynthesis pathway in the colon, thereby facilitating the agonist-induced secretion reaction of platelets and hemostatic platelet function [51]. In this study, platelets from GF mice presented reduced type I collagen-induced platelet activation compared to SPF controls and to mice colonized with spore forming bacteria [51]. Collagen-triggered granulophysin release, P-selectin surface expression, and exposure of activated integrin α_IIb_β_3_ on the platelet surface was found reduced in platelets from GF mice [51]. This data was also confirmed in the GF *Ldlr*^−/−^ mouse model under chow diet feeding conditions with adhesion-induced platelet deposition [48]. In addition, the meta-organismal TMAO pathway, which activation depends on choline-rich diet, was reported to promote arterial thrombus growth in the ferric chloride carotid artery thrombosis model [82]. This prothrombotic effect was explained by increased agonist-induced platelet reactivity in platelet-rich plasma and in isolated platelet suspensions, dependent on choline-rich diet feeding and plasma TMAO levels [82]. Zhu and coworkers reported that the exposure of platelets to physiologic TMAO concentrations is able to enhance submaximal thrombin-induced and ADP-triggered intracellular platelet Ca^2+^ levels. Other studies did not confirm a systemic impact of TMAO on coagulation in challenged mice (8 mg/kg of TMAO) [52]. A monocolonization study with *Clostridium sporogenes* and an isogenic deletion mutant elegantly demonstrated the influence of the gut microbial cutC TMA-lyase metabolism, the enzyme that converts choline to TMA, on arterial thrombus growth [88]. In the same study, interspecies fecal microbiota transplantation of a high-TMAO gut microbiota from human into GF mouse models demonstrated that TMAO plasma levels correlate with increased ADP-induced platelet aggregation and with reduced occlusion times in the ferric chloride carotid artery thrombosis model [88].

In addition to the mentioned influences on platelet functions, the presence of a gut microbiota also triggers a pro-adhesive phenotype in the vascular endothelium. GF mice show a reduced constitutive expression of intercellular adhesion molecule-1 (ICAM-1) in the liver and in other splanchnic organs compared to CONV-R and conventionalized mice [96]. Furthermore, studying a GF TLR2-deficient mouse model, we have recently shown that the presence of a gut microbiota stimulates VWF synthesis in the hepatic endothelium [17]. The microbiota-induced TLR2-mediated increase in plasma VWF levels, supported arterial platelet deposition in a ligation injury model of the common carotid artery, a mouse thrombosis model on platelet deposition to exposed subendothelial matrix molecules. A recent study by Kiouptsi et al. demonstrated the role of the gut microbiota in arterial thrombus formation in the *Ldlr*^−/−^ atherosclerosis model. In in vivo as well as ex vivo experiments on *Ldlr*^−/−^ mice, fed with a high-fat Western diet for sixteen-weeks, differences were noted when mice grew up under GF conditions. GF *Ldlr*^−/−^ mice presented lower leucocyte adhesion to the atherosclerotic vessel wall [48]. In conclusion, experiments on GF mouse models can give unique insights into the interplay between the gut microbiota and the host’s organ function, platelet reactivity and the immune system [17,48,95]. This technology is indeed one of the most meaningful experimental animal models, as it is complementary and reduces risks linked to the use of antibiotics in animal experiments [33].

## 5. Perspective

By the use of gnotobiotic mouse models, the gut microbiota was firmly linked to the onset of atherosclerosis and arterial thrombus growth. Based on these functional data, it will be most interesting to explore the functional changes in platelet physiology and endothelial cell biology that are provoked by colonization with a commensal microbiota and contribute to arterial thrombosis risk. Moreover, the identified bacterial species that were associated with an increased risk of atherosclerosis and arterial thrombosis should be tested with gnotobiotic rodent models for their functional impact on the regulation of prothrombotic pathomechanisms. Based on the wealth of experimental data, linking microbiota composition to arterial thrombosis, prospective clinical studies are needed and should consider including in-depth analyses of gut bacteria that are associated with an increased cardiovascular risk in order to explore their predictive diagnostic value.

## Figures and Tables

**Table 1:** Analyses on germ-free mouse atherosclerosis models.

Vessel Studied to Quantify Atherosclerosis	Diet/Feeding Duration	Age at Diet Switch	Reference
aortic root plaques	chow diet or western diet with and without 1.2% choline for 12 weeks	8 weeks	[14]
abdominal aorta plaques	chow diet and high-cholesterol western-type diet for 12–16 weeks	8 weeks	[15]
carotid artery plaques	high-cholesterol western diet (0.2% cholesterol) for 16 weeks	4–12 weeks	[48]
